# Classification of cervical neoplasms on colposcopic photography using deep learning

**DOI:** 10.1038/s41598-020-70490-4

**Published:** 2020-08-12

**Authors:** Bum-Joo Cho, Youn Jin Choi, Myung-Je Lee, Ju Han Kim, Ga-Hyun Son, Sung-Ho Park, Hong-Bae Kim, Yeon-Ji Joo, Hye-Yon Cho, Min Sun Kyung, Young-Han Park, Byung Soo Kang, Soo Young Hur, Sanha Lee, Sung Taek Park

**Affiliations:** 1 Department of Ophthalmology, Hallym University Sacred Heart Hospital, 22, Gwanpyeong-ro 170beon-gil, Dongan-gu, Anyang-si, Gyeonggi-do 14068 Republic of Korea; 2 Medical Artificial Intelligence Center, Hallym University Medical Center, Anyang, Republic of Korea; 3 Division of Biomedical Informatics, Seoul National University Biomedical Informatics (SNUBI), Seoul National University College of Medicine, Seoul, Republic of Korea; 4 Institute of New Frontier Research, Hallym University College of Medicine, Chuncheon, Republic of Korea; 5 Department of Obstetrics and Gynecology, Seoul St Mary’s Hospital, College of Medicine, The Catholic University of Korea, Seoul, Republic of Korea; 6 College of Medicine, Cancer Research Institute, The Catholic University of Korea, Seoul, Republic of Korea; 7 Department of Obstetrics and Gynecology, Hallym University Kangnam Sacred Heart Hospital, 1, Shingil-ro, Yeongdeungpo-gu, Seoul, 07441 Republic of Korea; 8 Department of Obstetrics and Gynecology, Hallym University Dongtan Sacred Heart Hospital, Hwaseong, Republic of Korea; 9 Department of Obstetrics and Gynecology, Hallym University Sacred Heart Hospital, Anyang, Republic of Korea

**Keywords:** Cervical cancer, Cancer screening, Gynaecological cancer

## Abstract

Colposcopy is widely used to detect cervical cancers, but experienced physicians who are needed for an accurate diagnosis are lacking in developing countries. Artificial intelligence (AI) has been recently used in computer-aided diagnosis showing remarkable promise. In this study, we developed and validated deep learning models to automatically classify cervical neoplasms on colposcopic photographs. Pre-trained convolutional neural networks were fine-tuned for two grading systems: the cervical intraepithelial neoplasia (CIN) system and the lower anogenital squamous terminology (LAST) system. The multi-class classification accuracies of the networks for the CIN system in the test dataset were 48.6 ± 1.3% by Inception-Resnet-v2 and 51.7 ± 5.2% by Resnet-152. The accuracies for the LAST system were 71.8 ± 1.8% and 74.7 ± 1.8%, respectively. The area under the curve (AUC) for discriminating high-risk lesions from low-risk lesions by Resnet-152 was 0.781 ± 0.020 for the CIN system and 0.708 ± 0.024 for the LAST system. The lesions requiring biopsy were also detected efficiently (AUC, 0.947 ± 0.030 by Resnet-152), and presented meaningfully on attention maps. These results may indicate the potential of the application of AI for automated reading of colposcopic photographs.

## Introduction

Cervical cancer is the fourth most common cancer in women worldwide, and the second most common cancer among females in developing countries^1^. Screening is the principal prevention method aimed at reducing mortality rates. Screening includes certain steps, including population-based Papanicolaou (Pap) testing, colposcopy-directed biopsy of suspicious lesions, and the treatment of confirmed pre-cancer lesions^2,3^. In women with low-grade intraepithelial lesions (LSIL) or high-grade intraepithelial lesions (HSIL), the risk of pre-cancer is medium to high, and immediate referral for colposcopy is necessary. However, referring all women with atypical squamous cells of undetermined significance (ASC-US) is considered inefficient, as the risk of such cases being pre-cancerous is lower^4^. Screening programs have been successful in the developed countries, leading to an approximately 80% decrease in the cervical cancer incidence over the past 4 decades. In contrast, the increase in cervical cancer incidence reported in developing countries^5^ has been attributed to the unsuccessful implementation of screening programs. This, has been attributed to logistics in health systems, infrastructural inadequacies, and the lack of expert physicians capable of introducing screening programs and follow-up^6^.

Colposcopy is an examination method, that identifies cervical lesions using low magnification microscopy under a strong light source^7^. It visualizes the cervical epithelium and facilitates the collection of cervical biopsy specimens for obtaining a histopathological diagnosis. Previous studies have evaluated the accuracy of colposcopic diagnoses and accompanying biopsies, finding a sensitivity of 70.9–98% and specificity of 45–90%^8–11^. However, the accuracy of colposcopic diagnosis is highly dependent on the physician’s skills, resulting in wide variability in its sensitivity and specificity between providers. This has raised concerns regarding the under-diagnosis of lesions, including missing cervical cancers^12^, or over-diagnosis of lesions, leading to over-treatment of low grade cervical lesions, increased risks of infections, patient discomfort, and financial burdens^13^.

Recently, artificial intelligence (AI) using machine learning has made considerable advances in medicine, allowing automated disease diagnosis based on medical image recognition^14,15^. Convolutional neural network (CNN), a kind of an artificial neural network, has shown excellent promise in reading fundus and skin photographs^16,17^. Machine learning has therefore been rapidly incorportaed in radiology, cardiology, gastroenterology, and even reproductive medicine^18–21^. Machine learning has already been introduced in colposcopic imaging; however, available evidence on its specificity and sensitivity is limited, preventing its full use in this field^22,23^.

This study had two aims. The primary aim was to develop a machine learning-based colposcopy model, that automatically classifies cervical neoplasms using two histopathologic systems: the classical cervical intraepithelial neoplasia (CIN) system and the lower anogenital squamous terminology (LAST) system^24^. The secondary aim of this study was to evaluate the performance of machine learning models in identifying cervical lesions requiring biopsies (neoplastic lesions vs. normal tissue). To our knowledge, this is one of the largest studies on the application of artificial intelligence to colposcopic photograph reading among biopsy-confirmed precancer cases.

## Materials and methods

### Study subjects

**Figure 1:**
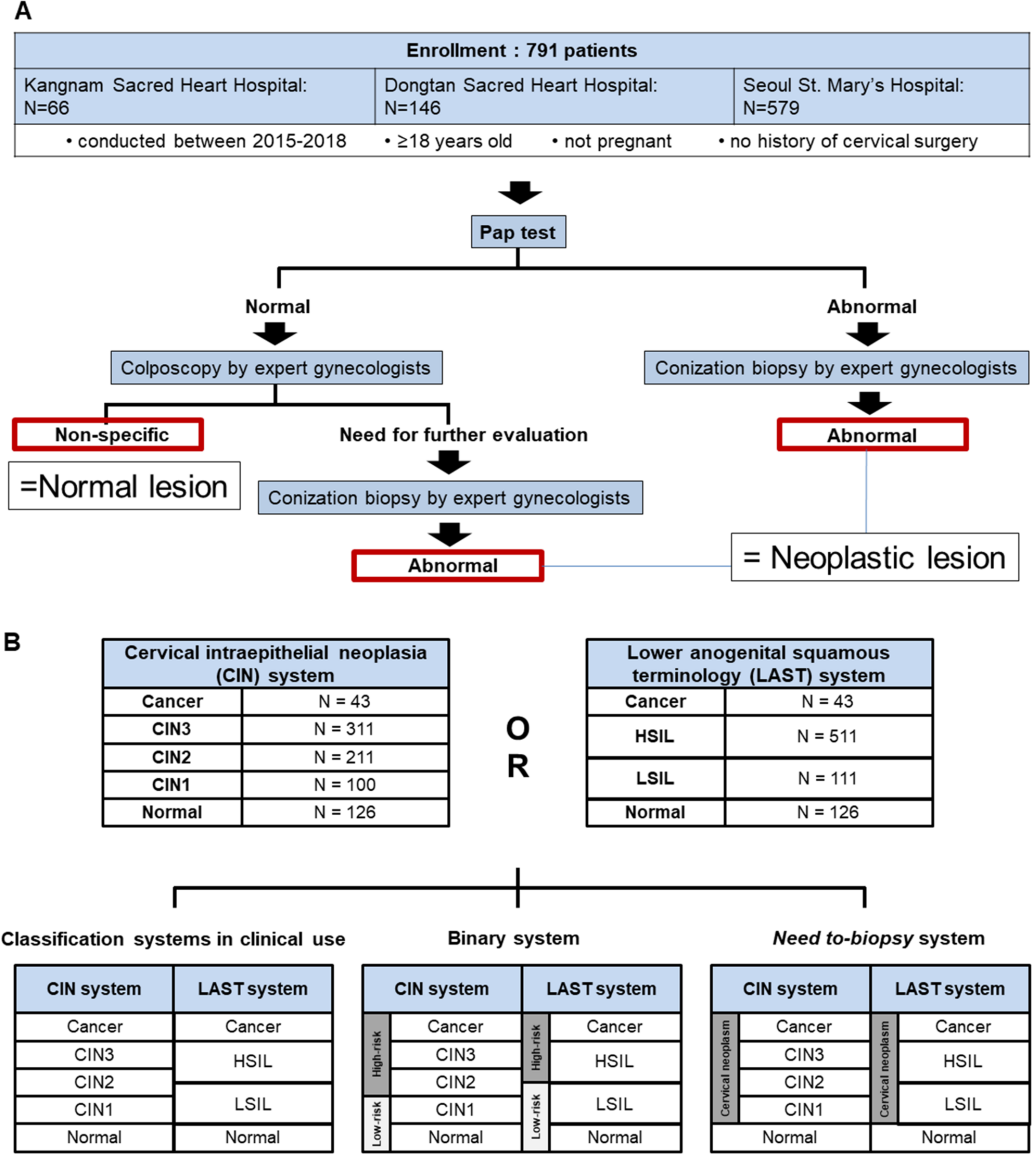
Participant enrollment diagram (**A**) and classification systems of cervical lesions (**B**).

Colposcopic photographs of cervical neoplastic or normal lesions, that were pathologically confirmed between 2015 and 2018, were retrospectively collected from three university-affiliated hospitals: the Kangnam Sacred Heart Hospital, Dongtan Sacred Heart Hospital, and Seoul St. Mary’s Hospital. Data eligible for inclusion in the study were from women who were ≥ 18 years old, not pregnant, had no history of cervical surgery, and had Pap test results. All neoplastic lesions were pathologically confirmed by conization biopsy, and normal lesions were defined as those with normal Pap test results, which were colposcopically normal, as confirmed by two gynecologic oncologists (S.T.P and Y.J.C) without pathologic (biopsy or conization) evaluation. In addition, all neoplastic lesions underwent human papillomavirus (HPV) testing (Fig. 1A and Table 1). This study was approved by the institutional review boards of the Kangnam Sacred Heart Hospital (IRB file number: 2018-01-031), Dongtan Sacred Heart Hospital (IRB file number: 2019-07-010), and Seoul St. Mary’s Hospital (IRB file number: KC18RESI0792) and complied with the principles of the Declaration of Helsinki. The need for informed consent was waived by the institutional review boards of the involved hospitals.

**Table 1:** Demographic characteristics of 791 women in this analysis.

Characteristics	No
**Age, y (n = 791)**	
18–29	139
30–49	451
50–94	201
**Pap result (n = 791)**	
Normal	159
ASC-US	147
AGC	13
ASC-H	109
LSIL	135
HSIL	223
Cancer	5
HPV result (n = 661)*	
Negative	46
Positive (Not HPV16)	425
HPV16	190

*Four women with cervical neoplasms did not have HPV results.

Binocular colposcopies with video monitoring systems were used for obtaining colposcopic photographs. After taking the photographs, colposcopy-directed biopsy and conization were performed by expert gynecologic oncologists with over 6000 cases of experience, according to the guidelines of the American Society for Colposcopy and Cervical Pathology^4^. All photographs were taken during colposcopic examination, before any operation or invasive procedure. After applying normal saline, the transformation zone and the region of interest were evaluated after being washed with 3–5% acetic acid. Only white-light images were used in the present study; these were retrieved from the picture archiving and communication systems of the participating hospitals with resolutions of 640 × 480 pixels. Images not allowing proper classification, such as poor quality or unfocused images, were excluded from the study; all personal identifiers were removed.

### Classification of cervical lesions

The collected images were reviewed by two gynecologic oncologists (S.T.P. and Y.J.C.), and only the image of the best quality was selected for each participant. Selected images were classified using two independent histopathologic systems: (1) the CIN system consisting of cervical cancer, CIN3, CIN2, CIN1, and normal, and (2) the LAST system including cervical cancer, HSIL, LSIL, and normal^24,25^.

Binary classification models dichotomizing cervical lesions into high-risk vs. low-risk lesions were then designed for each system. The CIN high-risk lesions included CIN2, CIN3, and cancer (CIN2+), and the CIN low-risk lesions included CIN1 and normal lesions (CIN1−). The LAST high-risk lesions included HSIL and cancer (HSIL+), and the LAST low-risk lesions included LSIL and normal lesions (LSIL−).

Lastly, a binary decision model was developed to determine the need to biopsy for a cervical lesion (Need-To-Biopsy system). The need to biopsy was defined as the lesion being classified as a neoplastic lesion by either the CIN or LAST system (CIN1+ or LSIL+). Therefore, the Need-To-Biopsy was defined as ‘not being normal’; therefore, there were only two classes in the Need-To-Biopsy system, namely, normal and Need-To-Biopsy, representing everything else except normal (Fig. 1B).

### Construction of datasets

The dataset was divided into training and test datasets, with a proportion of 85%: 15% by each class, as shown in Supplementary Table 1. This approach enabled testing of our model with the same ratio as the original dataset composition. After splitting the training and test datasets, data augmentation was performed for the training dataset to reduce class imbalance. Three different combinations of the training datasets were prepared using three different seed numbers for under-sampling, to check the robustness of the model. Lastly, the training set was further divided into the proper training dataset and the validation dataset for parameter tuning, at a ratio of 75:10. The datasets were mutually exclusive.

### Pre-processing of datasets

All images underwent automatic central cropping from the original resolution of 640 × 480 pixels to the new resolution of 480 × 480 pixels, removing 80 pixels for each right and left margin. All images were then normalized using min–max normalization to decrease the differences in photographs sourced from different participating hospitals.

For the training dataset, data augmentation was performed for the less frequent classes to overcome the data imbalance issue^25^. Data augmentation was customized for each training dataset built for each model by adding the rotated images of the training dataset. In the multi-class classification model for the CIN system, the cancer group was augmented six-fold by rotating the original images by 30°, 60°, 90°, 120°, and 150°. The CIN1 group was tripled by rotating the original images by 60° and 120°, and the normal group was doubled by rotating the original images by 90°. For the multi-class classification model of the LAST system, the normal and LSIL groups were quadrupled by rotating the original images by 45°, 90°, and 135°, and the cancer group was augmented tenfold. Finally, horizontal flipping, vertical flipping, and horizontal–vertical flipping were performed to augment the original data set four-fold.

### Training of the CNN models

Two CNN architectures were adopted, namely, the Inception-Resnet-v2 model (https://arxiv.org/abs/1602.07261) and Resnet-152 (https://arxiv.org/abs/1603.05027) model. In summry, the Resnet-152 is an updated version of the Resnet model, and the Inception-Resnet-v2 is a modified version of the Inception-v3 model, which incorporates some ideas adopted in the Resnet model. The CNN models were pre-trained by ImageNet weights and fine-tuned using the colposcopic images in this study.

Five different models were constructed for different labeling systems described above: multi-class CIN system, binary CIN system, multi-class LAST system, binary LAST system, and the need to biopsy. For binary classifications, new models were trained after creating datasets, which consisted of two classes. We did not simply induce the results from multi-class classifiers by converting the output into binary classes. Categorical cross-entropy was used as the loss function in the multi-class classification, and binary cross-entropy was used for binary classification. All training was performed using the PyTorch platform. Hardware systems were equipped with NVIDIA’s GeForce GTX 1080ti GPUs and dual Xeon central processing units.

The model training consisted of three stages, with images of a decreased resolution of 400 × 400 at the first stage, 450 × 450 resolution at the second stage, and 480 × 480 resolution at the last stage. Each stage consisted of two steps: (1) loading pre-trained models, unfreezing only the last layers, and training cyclically, and (2) unfreezing the entire layers and training cyclically with differential learning rates for the first few, middle, and last layers.

For each step, we performed the cyclic learning rate schedule proposed by Huang et al., but did not adopt the snapshot ensemble^26^. In summary, the initial learning rate was chosen at 1e-3, which presented the lowest validation loss in the single learning rate range test before initiating training^27^. The learning rate then followed a cosine annealing within one cycle, and returned to the initial learning rate at the start of the next cycle. Four cycles with a length of 1, 4, 16, and 64 were used in each step. In each cycle, early stopping was used to minimize validation loss. In performing step 2, three differential learning rates were used for different sublayers, and the initial learning rates were (1e−3)/9, (1e−3)/6, and 1e−3. Dropout was implemented with the dropout ratio of 0.5.

### Class activation map (CAM)

The attention map, or the CAM, was implemented to detect the region of interest^28^. For each CNN architecture, the last few layers were removed before a convolution layer was added, and global average pooling and softmax layers were applied. Multiplying feature maps spatially pooled using global average pooling for each corresponding class feature weight was performed to present the magnitude of importance in determining the class^28^. Up-sampling redirects to the localization in the original image. A class activation map was presented for each result using this method^28^. We selected red color to indicate the most activated region.

### Main outcome measures and statistical analysis

Class prediction of test datasets was performed with test-time augmentations (TTA) using four augmentations including original, horizontally-flipped, vertically-flipped, and horizontally–vertically-flipped images. The goal of the TTA was to increase the prediction accuracy by using the images from different perspectives. Four predictions were made for a single image; the average of the four predictions was taken as the final prediction.

To evaluate the model performances, three different training datasets with different seed numbers were used. Using the test dataset, which was not used for training, the multi-class classification accuracy was evaluated for the CIN and LAST criteria system. For binary classification, the area under the receiver operating characteristic curve (AUC) was calculated. Also, the mean accuracy, sensitivity, specificity, positive predictive value (PPV), and negative predictive value (NPV) were calculated at the optimal cutoff point, maximizing Youden’s index, the sum of sensitivity and specificity minus one. Continuous variables are expressed as means ± standard deviation. A *p* value of < 0.05 was regarded as statistically significant in all tests.

## Results

### Baseline characteristics

**Figure 2:**
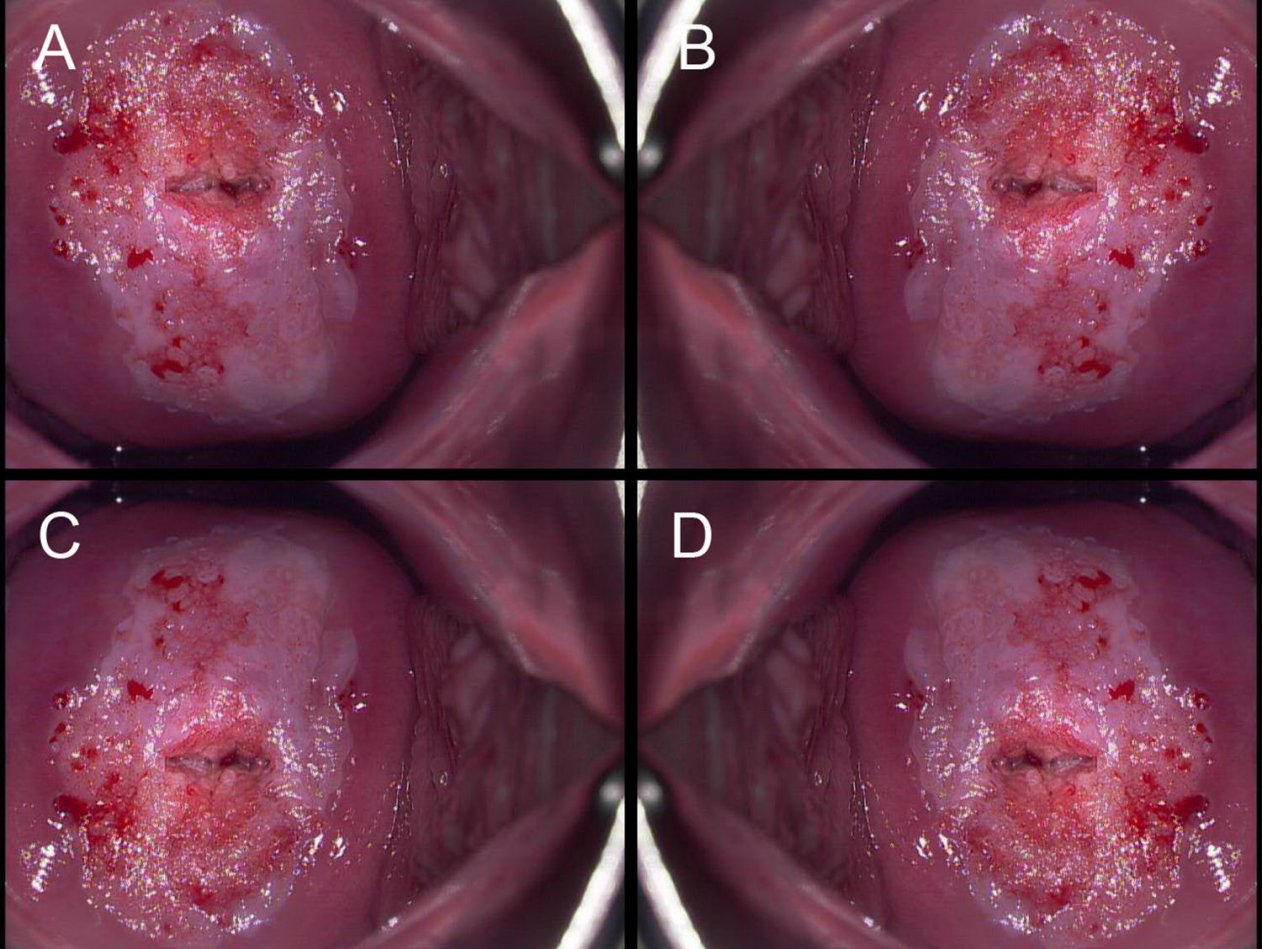
Representative examples of an original image (**A**) and the augmented images for cervical neoplastic lesion: a horizontally-flipped image (**B**), vertically-flipped image (**C**), and horizontally and vertically-flipped image (**D**).

A total of 1,426 images from 791 patients (1.8 images per each subject) were initially included, from which the images of the best quality were selected. Finally, 791 images from 791 patients were included in the study. The participant enrollment diagram and classification system used in this study are presented in Fig. 1. The entire dataset comprised 43 (5.4%) cancer images, 311 (39.3%) CIN3 images, 211 (26.7%) CIN2 images, 100 (12.6%) CIN1 images, and 126 (15.9%) normal images. In the LAST system, HSIL was the most frequent class (511, 64.6%), followed by normal. The data composition of the training and test datasets are shown in Supplementary Table 1. The test dataset comprised 116 images from 116 patients. One representative image is presented in Fig. 2. The associated augmented images are presented in Fig. 2B–D.

### Classification performances in the CIN system

**Figure 3:**
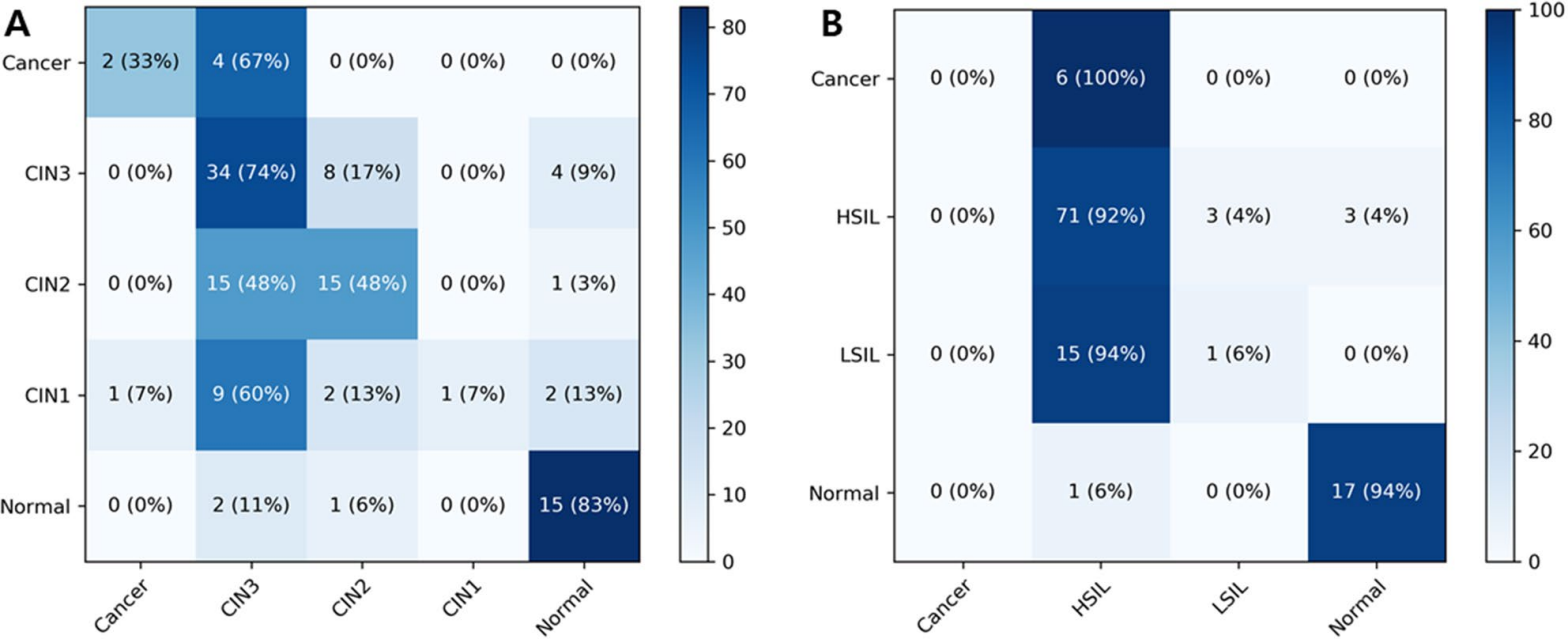
Heatmap of the confusion matrix of the multiclass classification of cervical lesions on colposcopic photographs by the best-performing Resnet-152 model. (**A**) the CIN system (**B**) the LAST system. The figure was created using Python version 3.6.8, sklearn library version 0.21.2 and matplotlip library version 3.1.0.

On classifying images into 5 categories by the CIN system, the mean overall accuracy of the CNN model in the test dataset was 48.6 ± 1.3% by the Inception-Resnet-v2 model and 51.7 ± 5.2% by the Resnet-152 model. On tenfold cross-validation, the accuracy of each model was 44.3 ± 2.1% by the Inception-Resnet-v2 model and 48.8 ± 1.9% by the Resnet-152 model. The mean per-class accuracies maximizing the Youden’s index for each class of the Resnet-152 model were 59.5 ± 9.7%, 56.6 ± 4.8%, 60.0 ± 6.1%, 57.2 ± 4.5%, and 92.8 ± 1.8% for cancer, CIN3, CIN2, CIN1, and normal lesions, respectively. The confusion matrix of the best performing Resnet-152 model is presented with a heatmap in Fig. 3A.

On binary classification for high- and low-risk lesions based on the CIN system, the mean AUC was 0.739 ± 0.024 by the Inception-Resnet-v2 model, and 0.781 ± 0.020 by the Resnet-152 model. The binary classification performances for the CIN, LAST, and the Need-To-Biopsy systems are presented in Table 2.

**Table 2:** Diagnostic performance of the machine learning models in the binary classification of cervical neoplasms on colposcopic photographs.

Model	Accuracy (%)	Sensitivity (%)	Specificity (%)	PPV (%)	NPV (%)	AUC
**High-risk lesions vs. Low-risk lesions in the CIN system**						
Inception-Resnet-v2	69.3 ± 4.8	66.7 ± 3.1	70.6 ± 6.1	47.2 ± 6.0	84.0 ± 1.8	0.739 ± 0.024
Resnet-152	68.9 ± 4.0	66.7 ± 3.1	69.9 ± 4.5	46.7 ± 5.0	84.2 ± 2.0	0.781 ± 0.020
**High-risk lesions vs. Low-risk lesions in the LAST system**						
Inception-Resnet-v2	63.2 ± 9.4	62.9 ± 7.6	63.5 ± 10.3	42.7 ± 9.3	79.9 ± 6.1	0.685 ± 0.072
Resnet-152	66.9 ± 3.4	65.7 ± 2.9	67.9 ± 3.7	46.1 ± 3.9	82.3 ± 2.0	0.708 ± 0.024
**Determining the need to biopsy**						
Inception-Resnet-v2	87.7 ± 0.5	83.3 ± 0.0	88.6 ± 0.6	57.0 ± 0.0	96.7 ± 0.0	0.932 ± 0.031
Resnet-152	87.7 ± 5.7	85.2 ± 3.2	88.2 ± 6.2	58.9 ± 15.4	97.0 ± 0.8	0.947 ± 0.030

*PPV* positive predictive value, *NPV* negative predictive value, *AUC* area under the curve, *CIN* cervical intraepithelial neoplasia, *LAST* lower anogenital squamous terminology.

### Classification performances in the LAST system

For the LAST system, the mean overall accuracy of 4-class classification was 71.8 ± 1.8% by the Inception-Resnet-v2 model and 74.7 ± 1.8% by the Resnet-152 model. The mean accuracy in tenfold cross-validation was 72.0 ± 4.5% by the Inception-Resnet-v2 model and 71.2 ± 4.9% by the Resnet-152 model. The mean per-class accuracies of Resnet-152 were 58.3 ± 13.4, 68.1 ± 0.9, 63.8 ± 7.9, and 91.7 ± 3.0 for cancer, HSIL, LSIL, and normal lesions, respectively. The CNN model was mostly effective at detecting normal and HSIL lesions (Fig. 3B).

The mean AUC for differentiating high-risk lesions from low-grade lesions in the LAST system was 0.685 ± 0.072 by the Inception-Resnet-v2 model and 0.708 ± 0.024 by the Resnet-152 model (Table 2). Notably, when only the Pap test results were used for the prediction of high-risk lesions, the mean AUC was 0.849 ± 0.014 in the CIN system and 0.827 ± 0.015 in the LAST system.

### Determining the need to biopsy

**Figure 4:**
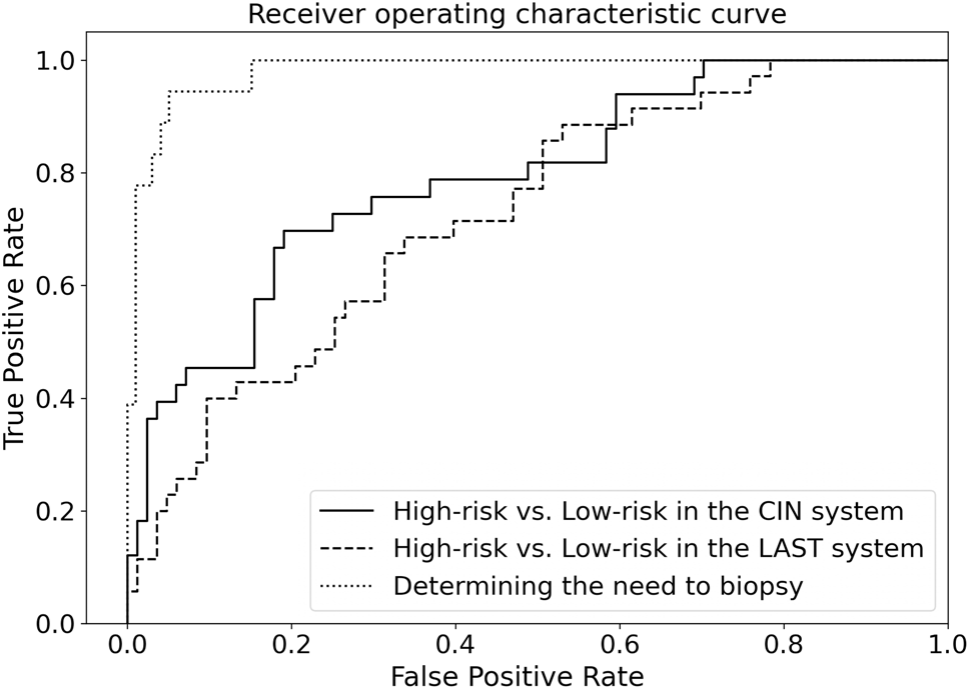
Receiver operating characteristic curves of the best-performing Resnet-152 models for the binary classification of the CIN and LAST system, and for determining the need to biopsy.

In determining the need to biopsy (‘Need-To-Biopsy System’), the mean AUC for determining the requirement for biopsy was 0.932 ± 0.031 and 0.947 ± 0.030 by the Inception-Resnet-v2 and Resnet-152 models, respectively (Table 2). The sensitivity and negative predictive value of the Resnet-152 model were 85.2 ± 3.2% and 97.0 ± 0.8%, respectively. The ROC curves of the best performing Resnet-152 models for differentiating high-risk lesions in the CIN and LAST system and for determining the requirement for biopsy, are presented in Fig. 4.

### Classification analyses

**Figure 5:**
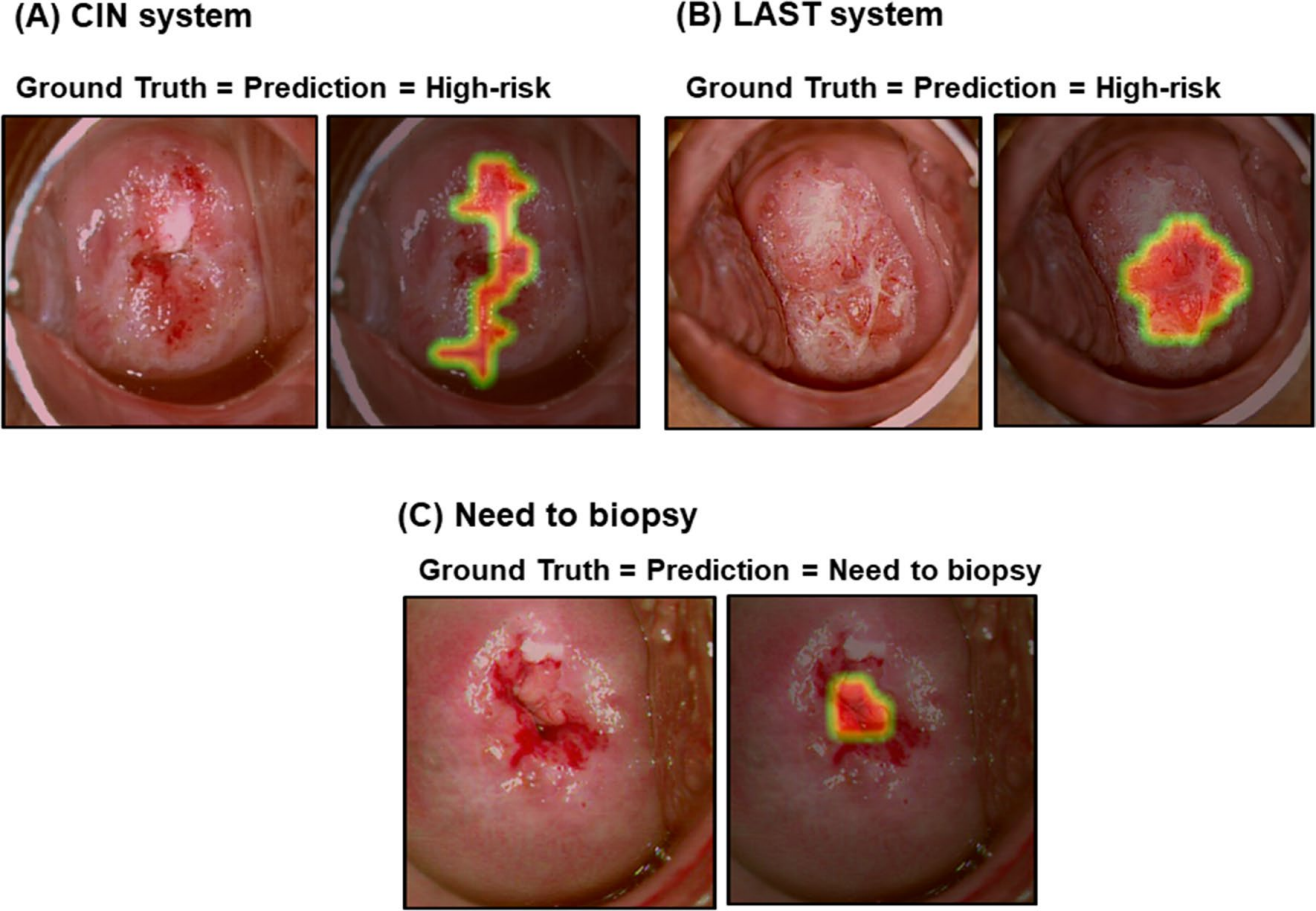
Class activation map for the classification of high-risk and low-risk cervical lesions on colposcopic photographs using a convolutional neural network based on (**A**) the CIN system or (**B**) the LAST system.

The CAM was reviewed for samples. Representative samples of CAM for high- and low-risk lesions are presented in Fig. 5. The deep learning model detected the suspicious area appropriately in most high-risk cases. In the review of CAM results by two gynecologic oncologists (S.T.P and Y.J.C), the CAM appropriately detected high-risk lesions judged as 82.5% for the CIN system, 89% for the LAST system, and 71.5% for the Need-To biopsy system.

## Discussion

Machine learning is considered promising in disease diagnosis and treatment-related decision-making, particularly in areas without enough medical experts with sufficient experience^29^. We investigated whether deep learning-based colposcopy can be used for disease diagnosis, and may lead to proper management decisions. In the present study, the mean accuracy for the CIN classification was 51.7 ± 5.2% by the Resnet-152 model; the mean AUC for differentiating high-risk (CIN2+) and low-risk (CIN1-) lesions reached 0.781 ± 0.020. The per-class accuracy of the deep learning model was 59.5 ± 9.7%, 56.6 ± 4.8%, 60.0 ± 6.1%, 57.2 ± 4.5%, and 92.8 ± 1.8% for cancer, CIN3, CIN2, CIN1, and normal tissue, respectively. For the LAST system, the mean accuracy of the Resnet-152 model was 74.7 ± 1.8%, and the mean AUC distinguishing high-risk (HSIL+) and low-risk (LSIL-) lesions was 0.708 ± 0.024. In addition, the mean AUC to determine the need for biopsy reached 0.947 ± 0.030. Collectively, these results suggest that machine learning-based colposcopy may be clinically applicable. The improved accuracy of the LAST system may be partly attributed to the fewer classes of the LAST system, as the classes in the CIN and LAST systems concur between systems, except that CIN2 may belong to either LSIL or HSIL.

The performance of human doctors in detecting cervical abnormalities on colposcopy have been reported to have 87–99% specificity and 26–87% sensitivity in previous studies^30^. For the purpose of this study, we simplified the currently used classification systems to distinguish cervical neoplasms from normal tissue. We developed a ‘Need-To-Biopsy System’ focusing on detecting neoplastic lesions which need biopsy. The performance of the deep learning model for this classification showed a mean AUC of 0.947, with 85.2% sensitivity and 88.2% specificity. This may suggest that the deep learning model may support under-experienced clinicians in deciding whether to perform a cervical biopsy or transfer the patient to a specialist. Notably, our Pap results provided better performance in detecting high-risk lesions than in previously reported results and our deep learning models. This may be partly explained by the fact that patients are expected to have inferior outcomes in tertiary institutions.

Till date, studies on classifying cervical lesions using CNN or other machine-learning algorithms on colposcopic images have been very limited. A previous study by Sato et al. showed that images were classified by the established CNN into three groups, namely, CIN3, carcinoma in situ (CIS), and invasive cancer (IC), with an overall accuracy of ~ 50%^22^. This study suggested that machine learning-based colposcopy may be clinically feasible, but did not provide satisfactory accuracy. Another study by Simoes et al. showed an accuracy of 72.15% in classifying colposcopic images with 170 image data. Although the accuracy was better than the average of previous studies, their study focused on technical issues in image classification without focusing on the clinical implications^23^. Recently, Hu et al. published a large-scale study deep learning based model using cervicography. The study showed a better accuracy (AUC = 0.91) in identifying CIN2+ cases^31^, compared to our data (AUC = 0.781). Hu et al. used a faster RCNN model, that first localizes the cervix and then classifies the lesion^31^. The localization step may have contributed to the improvement of performance. Nevertheless, the advantage of the faster R-CNN model might be reduced in analyzing colposcopy images, because the image only has a single region of interest (ROI), or the cervix, and the ROI is usually located centrally in most cases. Conversely, the CNN models we used (Resnet-152 and Inception-Resnet-v2) have been known to superior to the CNN model (VGG16) used by Hu et al. in the faster R-CNN. If a heavy model such as Resnet-152 was used as the backbone in the faster R-CNN, the performance might have been improved. Anyway, the differences in the performance between this study and the study by Hu et al.^31^ may be attributed to many factors including the localization process of the CNN model, CNN architecture, dataset size, data composition, and the image quality used in analyses.

Our machine learning model classified the cervical neoplasms according to two existing systems that are in current clinical use, namely, the CIN and LAST systems; between the two, the LAST system was established more recently to decrease the subjectivity of the histopathological classification, using the p16 biomarker, and to overcome the low reproducibility associated with the CIN system^25^. The LAST system is a 2-tiered system (HSIL and LSIL+ cancer and normal) and the CIN system is a 3-tiered system (CIN3, CIN2, and CIN1+ cancer and normal); their kappa statistic for reproducibility are 0.3–0.71 and 0.12–0.58, respectively^24^. The present study is the first to compare the machine learning model with the LAST system; previous studies used the machine learning colposcopy models only with the CIN system^22,23,31^.

In cases of suspected neoplastic lesions, distinguishing high- from low-risk lesions is important. In cases with low-risk cervical neoplasms, the cervical lesions may self-regress within 3 years. In contrast, most high-risk cervical neoplasms need treatment^32,33^. As the primary goal of screening is to distinguish between LSIL and HSIL, we developed a binary risk assessment system, that distinguished high-risk cervical neoplasms (CIN system, CIN2+ and LAST system, and HSIL+) and low-risk cervical neoplasms (CIN system, CIN1− and LAST system, and LSIL−). The mean AUC for differentiating high- from low-risk lesions was 0.781 ± 0.020 for the CIN-based system, and 0.708 ± 0.024 for the LAST-based system. To the best of our knowledge, only one previous report using deep learning for dichotomized classification of images from colposcopy has been published till date. That study used the CNN for classification of cancer vs. non-cancer images, and showed 83% diagnostic accuracy of the model^34^. However, that study used a different classification system from that system used in the present study; therefore, it is difficult to directly compare those results with the results from this study. Nevertheless, given that the purpose of screening is to detect early cervical neoplasms, we believe that the classification we used in the present study is more meaningful. Therefore, during colposcopic screening procedures, this algorithm may assist colposcopists to assess whether an ambiguous lesion requires biopsy. In the long-term, this approach may help prevent unnecessary biopsies.

The machine learning model used in this study has several strengths. First, the number of patients and images included in this study was the largest from all colposcopic machine learning models created till date. Previous studies have only included 51–158 subjects with 170–485 colposcopic images^22,23,35,36^, whereas our study included 791 subjects with 791 colposcopic images. Second, the colposcopic images were obtained from three medical centers (the Kangnam Sacred Heart Hospital, Dongtan Sacred Heart Hospital, and Seoul St. Mary’s Hospital). Third, our models attempted to reduce the false-positive rate by presenting the probability of lesions in all types of cervical neoplasms (based on the CIN and LAST systems), instead of providing a single definitive diagnosis. Moreover, binary classification with normal vs. neoplastic (requiring a biopsy) categories, and dichotomized classification with high-risk vs. low-risk classes could aid colposcopists accurately assess cervical lesions, and would help determine the necessity of a biopsy.

There are several limitations to our study. First, given the retrospective study design, there was a data imbalance, which may have induced unsatisfactory specificity. The high performance for CIN2 classification may be attributed to the class imbalance in the dataset. Although data augmentation using image rotation was tried for the minority classes, the ultimate data imbalance issue would have still remained. Second, owing to the multi-center design, there was heterogeneity in the image characteristics including contrast, brightness, tone, and quality among hospitals. Although we tried to normalize the images in the pre-processing stage, disparities may have persisted in the quality of images between hospitals. Third, in the automated central cropping process, part of the cervix may have been removed when it was located off-center in the image. Fourth, the limited data could partly account for the relatively poor performance. Our study is the largest to employ biopsy-confirmed application of artificial intelligence to colposcopic photographs; however, it is not adequate for comparing with other area artificial intelligence research, that showed good results. A prospective study including a large population is needed to address these issues.

## Conclusions

In summary, the proposed machine learning model classifying colposcopic images, reached performance levels comparable with those of experienced colposcopists, as assessed by previous studies. In addition, the deep learning model may support under-experienced clinicians in deciding whether to perform a cervical biopsy or transfer the patient to a specialist.

## Supplementary information

Supplementary file1 (DOCX 21 kb)

## References

[1] Torre L (2015). Global cancer statistics, 2012. CA Cancer J Clin.

[2] Wentzensen N (2017). Evidence-based consensus recommendations for colposcopy practice for cervical cancer prevention in the United States. J Low Genit Tract Dis.

[3] Wentzensen N, Schiffman M, Palmer T, Arbyn M (2016). Triage of HPV positive women in cervical cancer screening. J Clin Virol.

[4] Saslow D (2012). American Cancer Society, American Society for Colposcopy and Cervical Pathology, and American Society for Clinical Pathology screening guidelines for the prevention and early detection of cervical cancer. CA Cancer J Clin.

[5] Sankaranarayanan R (2014). Screening for cancer in low- and middle-income countries. Ann Glob Health.

[6] Torre L, Islami F, Siegel R, Ward E, Jemal A (2017). Global cancer in women: burden and trends. Cancer Epidemiol Biomark Prev (CEBP).

[7] Ji Q, Engel J, Craine E (2000). Texture analysis for classification of cervix lesions. IEEE Trans Med Imaging.

[8] Davies KR, Cantor SB, Cox DD, Follen M (2015). An alternative approach for estimating the accuracy of colposcopy in detecting cervical precancer. PLoS ONE.

[9] Massad LS, Jeronimo J, Katki HA, Schiffman M (2009). The accuracy of colposcopic grading for detection of high-grade cervical intraepithelial neoplasia. J Low Genit Tract Dis.

[10] Baldauf JJ, Dreyfus M, Ritter J, Philippe E (1997). An analysis of the factors involved in the diagnostic accuracy of colposcopically directed biopsy. Acta Obstet Gynecol Scand.

[11] Kierkegaard O, Byrjalsen C, Frandsen KH, Hansen KC, Frydenberg M (1994). Diagnostic accuracy of cytology and colposcopy in cervical squamous intraepithelial lesions. Acta Obstet Gynecol Scand.

[12] Stuebs FA (2019). Accuracy of colposcopy-directed biopsy in detecting early cervical neoplasia: a retrospective study. Arch Gynecol Obstet.

[13] Wentzensen N (2015). Multiple biopsies and detection of cervical cancer precursors at colposcopy. J. Clin. Oncol..

[14] Bi WL (2019). Artificial intelligence in cancer imaging: clinical challenges and applications. CA Cancer J Clin.

[15] Hosny A, Parmar C, Quackenbush J, Schwartz L, Aerts HJWL (2018). Artificial intelligence in radiology. Nat Rev Cancer.

[16] Gulshan V (2016). Development and validation of a deep learning algorithm for detection of diabetic retinopathy in retinal fundus photographs. JAMA.

[17] Esteva A (2017). Dermatologist-level classification of skin cancer with deep neural networks. Nature.

[18] Kallianos K (2019). How far have we come? Artificial intelligence for chest radiograph interpretation. Clin Radiol.

[19] Seetharam K, Shrestha S, Sengupta PP (2019). Artificial intelligence in cardiovascular medicine. Curr Treat Opt Cardiovasc Med.

[20] Cho BJ (2019). Automated classification of gastric neoplasms in endoscopic images using a convolutional neural network. Endoscopy.

[21] Wang R (2019). Artificial intelligence in reproductive medicine. Reproduction (Cambridge, Engand).

[22] Sato M (2018). Application of deep learning to the classification of images from colposcopy. Oncol Lett.

[23] Simoes PW (2014). Classification of images acquired with colposcopy using artificial neural networks. Cancer Inform.

[24] Waxman AG, Chelmow D, Darragh TM, Lawson H, Moscicki AB (2012). Revised terminology for cervical histopathology and its implications for management of high-grade squamous intraepithelial lesions of the cervix. Obstet Gynecol.

[25] Darragh TM (2012). The lower anogenital squamous terminology standardization project for HPV-associated lesions: background and consensus recommendations from the College of American Pathologists and the American Society for colposcopy and cervical pathology. Arch Pathol Lab Med.

[26] Huang, G. *et al.* Snapshot Ensembles: Train 1, get M for free. *arXiv e-prints* https://ui.adsabs.harvard.edu/abs/2017arXiv170400109H (2017).

[27] Simth, L. N. Cyclical learning rates for training neural networks. in *2017 IEEE Winter Conference on Applications of Computer Vision (WACV)*, 464–472 (2016).

[28] Zhou, B., Khosla, A., Lapedriza, A., Oliva, A. & Torralba, A. learning deep features for discriminative localization. *arXiv e-prints* https://ui.adsabs.harvard.edu/abs/2015arXiv151204150Z (2015).

[29] Rajkomar A, Dean J, Kohane I (2019). Machine learning in medicine. N Engl J Med.

[30] Mitchell MF, Schottenfeld D, Tortolero-Luna G, Cantor SB, Richards-Kortum R (1998). Colposcopy for the diagnosis of squamous intraepithelial lesions: a meta-analysis. Obstet Gynecol.

[31] Hu L (2019). An observational study of deep learning and automated evaluation of cervical images for cancer screening. J Natl Cancer Inst.

[32] Wright TC (2007). 2006 consensus guidelines for the management of women with cervical intraepithelial neoplasia or adenocarcinoma in situ. J Low Genit Tract Dis.

[33] McCredie MR (2008). Natural history of cervical neoplasia and risk of invasive cancer in women with cervical intraepithelial neoplasia 3: a retrospective cohort study. Lancet Oncol.

[34] Vasudha AM, Juneja M (2018). Cervix cancer classification using colposcopy images by deep learning method. Int J Eng Technol Sci Res.

[35] Asiedu MN (2019). Development of algorithms for automated detection of cervical pre-cancers with a low-cost, point-of-care, pocket colposcope. IEEE Trans Bio-Med Eng.

[36] Rouhbakhsh F, Farokhi F, Kangarloo K (2012). Effective Feature Selection for Pre-Cancerous Cervix Lesions Using Artificial Neural Networks. Int J Smart Electr Eng.

