# Supplementary material for: Classification of cervical neoplasms on colposcopic photography using deep learning
Source: Sci Rep. 2020 Aug 12;10:13652. doi: 10.1038/s41598-020-70490-4 (PMC7423899; doi:10.1038/s41598-020-70490-4)
Supplement: Supplementary file 1 — Supplementary file1 (DOCX 21 kb) [file 41598_2020_70490_MOESM1_ESM.docx]

**Manuscript title:**

**Classification of cervical neoplasms on colposcopic photography using deep learning**

**Author list:**

Bum-Joo Cho MD^1,2,3,4,*,#^; Youn Jin Choi MD ^5,6,*^; Myung-Je Lee BS^2^; Ju Han Kim MD^3^; Ga- Hyun Son MD ^4,7^; Sung-Ho Park MD ^7^; Hong-Bae Kim MD ^7^; Yeon-Ji Joo MD ^7^; Hye-Yon Cho MD ^8^; Min Sun Kyung MD ^8^; Young-Han Park MD ^9^; Byung Soo Kang MD^5^; Soo Young Hur MD^5,6^; Sanha Lee MD^5,6^; Sung Taek Park MD^4,7,#^

^*^These two authors equally contributed to this work as the first authors.

^#^These two authors equally contributed to this work as the corresponding authors.

**Author’s Affiliations**

^1^Department of Ophthalmology, Hallym University Sacred Heart Hospital, Anyang, Korea

^2^Medical Artificial Intelligence Center, Hallym University Medical Center, Anyang, Republic of Korea

^3^Interdisciplinary Program in Medical Informatics, Seoul National University College of

Medicine, Seoul, Korea

^4^Institute of New Frontier Research, Hallym University College of Medicine, Chuncheon, Korea

^5^Department of Obstetrics and Gynecology, Seoul St Mary’s Hospital, College of Medicine, The Catholic University of Korea, Seoul, Republic of Korea

^6^Cancer Research Institute, College of Medicine, The Catholic University of Korea, Seoul, Republic of Korea

^7^Department of Obstetrics and Gynecology, Hallym University Kangnam Sacred Heart Hospital, Seoul, Korea

^8^Department of Obstetrics and Gynecology, Hallym University Dongtan Sacred Heart Hospital, Hwaseong, Korea

^9^Department of Obstetrics and Gynecology, Hallym University Sacred Heart Hospital, Anyang, Korea

**Corresponding author:**

**Sung Taek Park, MD, PhD**

Department of Obstetrics and Gynecology, Hallym University Kangnam Sacred Heart Hospital, 1, Shingil-ro, Yeongdeungpo-gu, Seoul 07441, Republic of Korea.

Tel: +82-2-829-5114, Fax: +82-2-829-5134, E-mail: parkst96@gmail.com

**Bum-Joo Cho, MD, PhD**

Department of Ophthalmology, Hallym University Sacred Heart Hospital, 22, Gwanpyeong-ro 170beon-gil, Dongan-gu, Anyang-si, Gyeonggi-do 14068, Republic of Korea.

Tel: +82-31-380-3835, Fax: +82-31-380-3837, E-mail: bjcho8@gmail.com

Supplementary table 1. Data composition of enrolled colposcopic photographs in each dataset for multi-class classification

|  | | Entire dataset | | | | Training dataset | Test dataset |
| --- | --- | --- | --- | --- | --- | --- | --- |
|  |  | Total | Kangnam | Dongtan | St. Mary’s |  |  |
| **Overall** | | 791 | 66 | 146 | 579 | 675 | 116 |
| **CIN system** | Normal | 126 | 0 | 0 | 126 | 108 | 18 |
|  | CIN1 | 100 | 6 | 55 | 39 | 85 | 15 |
|  | CIN2 | 211 | 28 | 26 | 157 | 180 | 31 |
|  | CIN3 | 311 | 28 | 46 | 237 | 265 | 46 |
|  | Cancer | 43 | 4 | 19 | 20 | 37 | 6 |
| **LAST system** | Normal | 126 | 0 | 0 | 126 | 108 | 18 |
|  | LSIL | 111 | 6 | 55 | 50 | 95 | 16 |
|  | HSIL | 511 | 56 | 72 | 383 | 435 | 76 |
|  | Cancer | 43 | 4 | 19 | 20 | 37 | 6 |

CIN = cervical intraepithelial neoplasia, LAST = lower anogenital squamous terminology, LSIL = low-grade intraepithelial lesion, HSIL = high-grade intraepithelial lesion
